# Autograft and allograft bone chips interbody fusion for spondylodiscitis: Surgery outcomes

**DOI:** 10.22088/cjim.14.1.133

**Published:** 2023

**Authors:** Majid Rezvani, Amirhosein Zohrevand, Parisa Azimi, Soheil Fallahpour, Saeid Saghaei, Taravat Yazdanian, Mohammadjavad Pashnehtalaee

**Affiliations:** 1 Department of Neurosurgery, School of Medicine, Neuroscience Research Center, Al-Zahara Hospital, Isfahan University of Medical Sciences, Isfahan, Iran; 2 Department of Neurosurgery, Isfahan University of Medical Sciences, Isfahan, Iran; 3 Neuroscience Research Center, Shahid Beheshti University of Medical Sciences, Tehran, Iran; 4 School of Medicine, Capital Medical University, Beijing, China; 5 Faculty of Medicine, Isfahan University of Medical Sciences, Isfahan, Iran

**Keywords:** Spondylodiscitis, Modified interbody fusion, JOABPEQ, ASIA, surgery

## Abstract

**Background:**

Spondylodiscitis is a rare illness and serious complication of the vertebral column. The suitable type of surgery is debatable for these patients. This study describes a series of cases that are treated with modified interbody fusion for the treatment of spondylodiscitis by combining allograft and autograft bone chips with posterior segmental fusion.

**Methods:**

This was a retrospective study. The clinical deficit was evaluated with ASIA, VAS, and JOABPEQ scores before and after surgery. Radiological parameters were assessed with local kyphosis angle (degree), segmental height correction, and loss of correction. Post-operative bone union was evaluated as suggested by Tan et al.

**Results:**

The mean age of patients (n=34) was 52.3(SD=13.6) years and 67.6% were males. The mean follow-up duration was 25.8 (2.3) months. In the last follow-up, VAS back pain 4.9(0.77), VAS leg pain 4.6(0.78), JOABPEQ low back pain 68.1 (9.3), JOABPEQ lumbar function 81.3 (8.9), and JOABPEQ walking ability 72.8 (8.3) shows a significant difference when compared with preoperative scores. According to ASIA grading, none of the patients deteriorated neurologically (all p<0.0001). The average segmental height correction and loss of correction were observed 7.5(3.7) % and -1.8(3.6) %, respectively, indicating improvements in the patients. A high union fusion rate (82.4%) was observed in the last follow-up.

**Conclusion:**

This modified method can be a safe and effective technique for surgical intervention in patients with spondylodiscitis.

Spondylodiscitis is a condition that involves the intervertebral disc space and adjacent vertebrae by infective process (1). It is a complex and multifactorial illness, whose diagnosis and treatment are remained unclear (1-2). The main pillar of treatment for spondylodiscitis tends to be conservative; bed rest, involving antibiotics, and optimal spinal stabilization. Surgery is performed when medical management fails or cases present with the instability of the spine or compromise the nerve root function (2). For treating spondylodiscitis, several surgical procedures such as anterior-only, posterior-only, and combined anterior-posterior with different types of interbody fusion has been proposed, nonetheless, the optimal surgical treatment remains debatable (2). Each interbody fusion has its advantages and disadvantages. Hence, a new surgery technique or modified interbody fusion is needed to reduce complications. We aimed to describe our experiences with operative treatment on spondylodiscitis with the combination of autograft and allograft bone chips interbody fusion. It will also discuss the clinical summary, surgical procedures, and outcomes.

## Methods

Ethical approval was obtained from the Research Ethics Committee of the Isfahan University of Medical Sciences (ref nr: IR.MUI.MED.REC.1400.567).

This was a retrospective study. It was conducted on 34 patients with spondylodiscitis who had undergone posterior lumbar/thoracic interbody fusion using compressive bone graft with the combination of autograft and allograft bone chips at a single medical center from December 2018 to October 2021. All surgeries were performed by a single surgeon. Data were collected through a review of patient records and relevant imaging. The diagnosis was according to clinical findings, radiological imaging, and laboratory results (3). 

All patients included in this study had a trial of conservative management, and they were only considered for spine surgery after failure of conservative treatment. The inclusion criteria consisted of age >18 years with vertebral body distraction less than 40% of body height, regularly followed for at least 24 months. Patients with severe osteoporosis and upper T5 level involvement were excluded.

**Surgical technique**: Once a diagnosis of spondylodiscitis had been clinically established, patients underwent posterior approach surgery. After pedicle screws insertion, laminectomy and partial facetectomy were performed. Infected disc, endplates, and distracted vertebral body were removed. Allograft cancellous and autograft (spinous process) bone chips were impregnated with one gram of vancomycin powder. After mild distraction, it was inserted in the interbody space and impacted under the intraoperative neuromonitoring recording. Then compression was applied to increase bone-bone interface and further correction of spinal alignment. After fixation of rods and posterolateral fusion, the wound was closed in the traditional layer manner.

**Measures: **(I) Clinical outcomes were evaluated by visual analogue scale (VAS) (4) for back/leg pain, and American Spinal Injury Association (ASIA) Impairment score (5) for neurological assessment. ASIA impairment scale for classifying spinal cord injury includes A= Complete injury; B= Sensory incomplete; C= Motor incomplete with a muscle grade less than 3; D= Motor incomplete with a muscle grade >3; and E= Normal. (II) The Japanese Orthopedic Association Back Pain Evaluation Questionnaire (JOAPEQ) score: The JOABPEQ is a disease-specific tool for low back pain and contains 25 items tapping into five subscales: social function (4 items), mental health (7 items), lumbar function (6 items), walking ability (5 items), and low back pain (4 items). The score for each subscale ranges from 0 to 100, with higher scores indicating better conditions (6). The JOABPEQ subscale scores were calculated at baseline and the last follow-up surgery. In this study, low back pain, lumbar function, and walking ability were considered. (III) The radiological assessment included segmental height (mm), local kyphosis angle (degree), segmental height correction, loss of correction, and fusion rate. Post-operative bone union was evaluated based on grading of allograft/autograft interbody fusion using CT, as suggested by Tan et al. (7), was categorized as follows: Grades I and II were considered evidence of fusion or impending fusion. Grades III and IV were taken as evidence of nonunion.

Clinical and radiographic (MRI, CT, and plain radiographs) investigations were conducted preoperatively, early postoperatively, and late postoperatively (last follow- up). Statistical analysis of the data was performed using PASW Version 18 (SPSS Inc., Chicago, IL, USA). For continuous values, the mean was considered to derive the central tendency of the data. Comparison of ASIA scales before surgery and the final follow-up were assessed by χ2 test. Grouped values were evaluated using a Pearson chi-square test; values of ≤ 0.05 were considered significant.

**Ethics: **The Ethics Committee of Isfahan University of Medical Sciences, Isfahan, Iran, approved the study.

## Results

**Figure 1:**
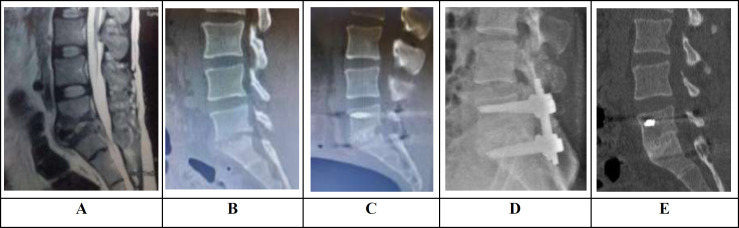
Sagittal T2W lumbosacral MRI (A) and sagittal lumbosacral CT (B) showed preoperative findings of spondylodiscitis at L5-S1. The operation was carried out with posterior instrumentation and allograft autograft combination bone chips interbody fusion at L5-S1 level (C). At 25 months after surgery, lateral X-ray (D), and sagittal lumbosacral CT (E) showed rigid fixation and fusion without any evidence of pseudoarthrosis

Characteristics of patients are shown in table 1. All patients were followed-up for at least 24 months postoperatively. The average clinical follow-up was 25.8 (SD= 2.3) months. Our clinical assessment compared patients' VAS, JOABPEQ, and ASIA scores, before and after surgery. The results are shown in table 2. The rigid bony union was observed in all patients at the last follow-up (figure 1).

**Table 1:** Characteristics of patients with spondylodiscitis (n=34)

**Characteristics**	
Age (year)	52.3(13.6)
Gender (male %)	23(67.6)
BMI (kg/m2)	23.5(3.5)
**Level of infection**	
Thoracic, n (%)	4 (11.8)
Thoracolumbar, n (%)	3 (8.8)
Lumbar, n (%)	20(58.8)
Lumbosacral, n (%)	7(20.6)

*Values are mean (SD) or percentage.

Regarding pain and functional improvement, the average change in VAS score and JOABPEQ subscales scores shows a significant difference when compared with preoperative scores (all p<0.0001). According to the ASIA scale criteria, 16 (47.1%) patients and 14 (41.2%) patients had Grade D and Grade E, respectively (table 2). No case reported worse neurological findings postoperatively. The average segmental height correction and loss of correction were observed 7.5 (3.7) % (range: 0 – 15%) and - 1.8 (3.6) % (range: - 20 – 0.0%), respectively. The most common risk factors for infection included uncontrolled type 2 diabetes mellitus, pneumonia, chronic renal failure (CRF), urinary tract infection (UTI), and corticosteroid therapy. In our study, 7 (20%) developed postoperative complications, out of which 4 developed deep vein thrombosis (DVT) and 3 were found to have a superficial wound infection. None of the patients needed reoperation.

**Table 2:** Clinical outcomes of surgical treatment for spondylodiscitis (n=34)

	**Pre-operative**	**Post-operative**		**P-value**
		**Early post-operative**	**Last follow-up**	
**Local Kyphosis angle (degree)**				
Thoracic	8.5(2.4)	4.3(3.6)	4.3(3.8)	0.111
Thoracolumbar	5.3(1.5)	-2.3(1.5)	-1.7(0.6)	0.002
Lumbar/ Lumbosacral	-7.2(6.8)	-11.6(6.2)	-10.5(5.9)	0.062
**ASIA score**				< 0.0001
Grade B, n (%)	11(32.4)	-	0	
Grade C, n (%)	14(41.2)	-	4(11.8)	
Grade D, n (%)	4(11.8)	-	16(47.1)	
Grade E, n (%)	5(14.7)	-	14(41.2)	
**VAS score Leg**	9.7(0.49)	4.3(0.85)	4.6(0.78)	< 0.0001
**VAS score Back**	9.8(0.55)	4.4(1.11)	4.9(0.77)	< 0.0001
**JOABPEQ**				
Low back pain	36.4 (24.5)	-	68.1 (9.3)	< 0.0001
Lumbar function	41.8 (24.9)	-	81.3 (8.9)	< 0.0001
Walking ability	40.2 (29.9)	-	72.8 (8.3)	< 0.0001
**Grade of fusion**				
Grade 1, n (%)	-	-	4 (11.8)	-
Grade 2, n (%)	-	-	24 (70.6)	-
Grade 3, n (%)	-	-	4 (11.8)	-
Grade 4, n (%)	-	-	2 (5.9)	-

*Values are mean (SD) or percentage; VAS, visual analogue scale; American Spinal Injury Association (ASIA) Impairment score including: A = Complete injury; B = Sensory incomplete; C = Motor incomplete; D = Motor incomplete; and E = Normal.; JOAPEQ, Japanese Orthopedic Association Back Pain Evaluation Questionnaire; Grade of fusion, interbody fusion using CT, as suggested by Tan et al. (7).

## Discussion

To our knowledge, we reported the first case series of patients with spondylodiscitis undergoing allograft and autograft combination bone chips interbody fusion with the posterior approach. This is a good technique to obtain spine pain relief, improve functional outcomes, and have good bone union with low complication rates.

The average change in VAS score for back pain was 4.9, while that for leg pain was 5.1. Our study results are comparable with the results of Zhao et al. (8), which means VAS changes were significant. Regarding functional improvement, the average change in JOABPEQ subscales scores was low back pain 31.7, lumbar function 39.5, and walking ability 32.6, which shows a significant difference when compared with preoperative scores (all p<0.0001). In the literature, we did not find any study to assess outcomes of surgery for patients with spondylodiscitis based on JOABPEQ. Zhao et al. (8), reported that according to ASIA grading, none of the patients deteriorated neurologically after surgery treatment, which is in line with our finding.

Pee et al. (9), was presented that successful bone union was achieved 91% and 97% in the iliac strut group and the cage group, respectively. In similar studies carried out by Zhao et al. (8), and Lin et al. (10), satisfactory bone fusion was achieved in all patients. However, in the abovementioned studies, the assessment of bone union was according to postoperative plain x-ray or CT without specific criteria. Overall, several studies have highlighted mixed results to the bone union, due to different diagnostic criteria. In this study, bone union was evaluated based on the Tan et al. scale (7), which seems to be a more accurate method. Only 2 patients presented with a poor bone union (grade 4 on the Tan et al. scale), due to comorbidities such as uncontrolled type 2 DM, CRF, and corticosteroid therapy.

Spine infection normally affects the anterior vertebral elements, and an anterior surgical technique is recommended to debride the infected material. However, anterior spinal surgery is limited by the perceived seriousness of the potential complications, in particular major vascular injury. The posterior approach is recommended, as this has more advantages compared to the anterior approach, and faster fusion attained with posterior instrumentation (11). In this study, the posterior approach includes debridement, decompression, anterior column reconstruction, and instrumentation was applied. The rate of loss of correction was low because of acceptable anterior column reconstruction and fusion. The results were similar to the study of Sundararaj et al. (12). Titanium and polyetheretherketone (PEEK) cages have been assessed in the lumbar spine, with conflicting outcomes in bony fusion and subsidence. A meta-analysis was reported that titanium and PEEK cages were associated with a comparable rate of fusion, but there is an increased rate of subsidence with titanium cage (13). In addition, there still existed the concern of bacterial biofilm formation during the use of the PEEK cages (14) and the titanium cages (15) at the site of the infection. The autograft provides both osteoconductive and osteoinductive properties but with associated donor site morbidity and increased operative time. Though the allograft is associated with a higher risk of nonunion (12). In this study, a combination of the allograft and the autograft bone was used to achieve the best surgical outcomes compared to other techniques. We reconstructed the spinal defect with small bone chips without any pressure or damage to the nerve roots.

The current study had some limitations. First, the low sample of patients should be addressed in further studies. Second, a new technique may or may not be better than an existing one and a clinical trial is can be performed to find the best technique. Third, due to biomechanical problems, this method is not considered for patients with more than 40% vertebral body height involvement. Other surgical methods such as titanium or mesh cages can be recommended. However, strengths of the study include a long-term follow-up study was presented of 34 cases with spondylodiscitis undergoing interbody fusion surgery. Additionally, titanium cages as PEEK cages are expensive compared to this method. Finally, our experience demonstrates that this method can significantly improve clinical outcomes.

This study shows that this technique can result in a successful bone union of the spine, improved pain assessment, and neurological outcomes in patients with spondylodiscitis. Future prospective randomized controlled trials are required to further evaluate this method using surgical and patient-reported tools.
